# Gamma tACS over the temporal lobe increases the occurrence of *Eureka!* moments

**DOI:** 10.1038/s41598-019-42192-z

**Published:** 2019-04-08

**Authors:** Emiliano Santarnecchi, Giulia Sprugnoli, Emanuela Bricolo, Giulio Costantini, Sook-Lei Liew, Christian S. Musaeus, Carola Salvi, Alvaro Pascual-Leone, Alessandro Rossi, Simone Rossi

**Affiliations:** 1 Berenson-Allen Center for Non-Invasive Brain Stimulation, Department of Neurology, Beth Israel Deaconess Medical Center, Harvard Medical School, Boston, MA USA; 2 Brain Investigation & Neuromodulation Laboratory (Si-BIN Lab), Department of Medicine, Surgery and Neuroscience, Unit of Neurology and Clinical Neurophysiology, University of Siena, Siena, Italy; 3 Psychology Department, University of Milano-Bicocca, Milan, Italy; 4 Milan Center for Neuroscience, Milan, Italy; 5 Chan Division of Occupational Science and Occupational Therapy, University of Southern California, Los Angeles, CA USA; 6 Department of Neurology, Danish Dementia Research Centre (DDRC), Rigshospitalet, University of Copenhagen, Copenhagen, Denmark; 7 Northwestern University, Psychology department, Evanston, IL USA; 8 Rehabilitation Institute of Chicago, Chicago, IL USA; 9 Human Physiology Section, Department of Medicine, Surgery and Neuroscience, University of Siena, Siena, Italy

## Abstract

The solution to a problem might manifest itself as a burst of unexpected, unpredictable clarity. Such *Eureka!* events, or Insight moments, are among the most fascinating mysteries of human cognition, whose neurophysiological substrate seems to include a role for oscillatory activity within the α and γ bands in the right parietal and temporal brain regions. We tested this hypothesis on thirty-one healthy participants using transcranial Alternating Current Stimulation (tACS) to externally amplify α (10 Hz) and γ (40 Hz) activity in the right parietal and temporal lobes, respectively. During γ-tACS over the right temporal lobe, we observed an increase in accuracy on a verbal insight task. Furthermore, electroencephalography (EEG) data revealed an increase in γ spectral power over bilateral temporal lobes after stimulation. Additionally, resting-state functional MRI data acquired before the stimulation session suggested a correlation between behavioral response to right temporal lobe tACS and functional connectivity of bilateral temporal lobes, in line with the bilateral increase in γ band revealed by EEG. Overall, results suggest the possibility of enhancing the probability of generating *Eureka!* moments in humans by means of frequency-specific noninvasive brain stimulation.

## Introduction

Insight problem-solving occurs when the solution to a problem comes to one’s awareness in a sudden and unexpected *Eureka!* (or *Aha!*) moment. It has been described as “a great speculative leap” (Einstein in^1^) and it cannot be explained with a continuous sequence of reasoning steps. Research on insight problem-solving began about a century ago with Köhler’s observations on the problem-solving abilities of chimpanzees^2^. During the last two decades, multiple theories and models about the neurobiology of insight have been proposed, including: (i) the Criterion for Satisfactory Progress theory (formerly known as Progress Monitoring Theory^3^), (ii) the Special-Process Theory^4^, and (iii) the three-steps model proposed by Beeman, Kounios and Bowden^5–7^. The latter has gained particular credit thanks to neuroimaging and electrophysiological support^8^. According to this theory, three steps lead to an insight moment: (i) a strong activation of irrelevant consolidated knowledge coupled with a weak activation of new information; (ii) a secondary integration and reorganization of information; (iii) the rise of a new pattern (*i.e*., the solution) to consciousness^7^. Interestingly, this model includes spatially and temporally distributed inter-hemispheric activity, which has been corroborated by experimental evidence including e.g. studies using visual-hemifield presentation of insight problem-solving tasks^9^. Overall, insight-related processing is supposed to rely on a first coarse semantic coding taking place in the right hemisphere, which weakly and diffusely activates alternative meanings and potential interpretations of the stimuli at hand. At the same time, processing in the left hemisphere supports fine semantic coding, promoting stronger neural activity for a single dominant interpretation and only a few close or contextually appropriate alternatives^10–13^. In this context, left hemisphere fine semantic coding have a clear advantage for the comprehension of direct language, while right-hemisphere activity is necessary for the comprehension of “indirect” language such as jokes, metaphors, inferences and insightful solutions (for a review, see^14^).

**Figure 1:**
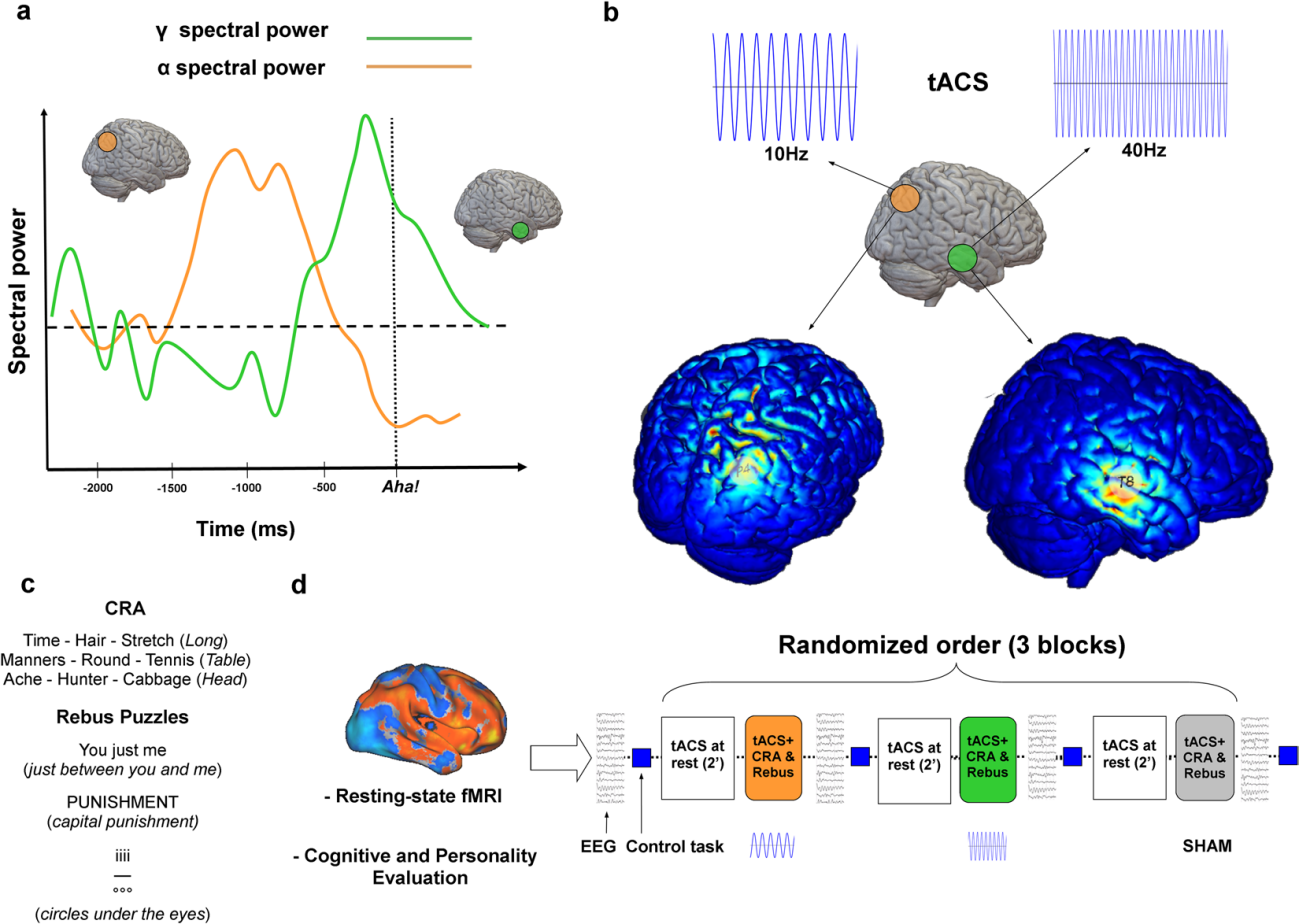
Experimental design. (**a**) A graphical representation of the theoretical model of insight problem-solving proposed by Jung-Beeman and colleagues^8^, showing the contribution of brain oscillations in the α and γ bands before a *Eureka!* moment, with α activity in the right parietal lobe (orange circle) preceding a burst of γ band in the right temporal pole (green circle). (**b**) The role of α oscillations at an early stage, and γ ones right before the successful solution of a given problem, constituted the rationale for the adopted brain stimulation solutions. The detailed modeling of the electrode montage and induced electric field are provided in Fig. S1 and Supplementary Information. (**c**) Given prior EEG and fMRI evidence, electrical stimulation was delivered over the right parietal and temporal lobes, respectively at 10 Hz and 40 Hz (continuously for each block of stimulation), while participants solved a verbal (CRA) and a visuo-spatial (Rebus Puzzles) insight task. (**d**) EEG data were recorded before and after each stimulation block (i.e. Sham, 10 Hz tACS on right parietal lobe, 40 Hz on right temporal area). Cognitive and resting-state fMRI data were also collected on a separate day to investigate possible sources of variability in the individual response to tACS (see Supplementary Information).

In line with studies reporting the importance of the right anterior superior temporal gyrus (rSTG) in the creation of distant semantic relations^15,16^, Beeman and colleagues^8^ have documented increased functional magnetic resonance imaging (fMRI) activity in the rSTG for solutions achieved via insight processes, as well as a burst of gamma (γ; ~40 Hz) oscillatory activity occurring in the same cortical region 300 ms before a correct, insight-based solution (using electroencephalography – EEG – recording). In addition, immediately prior to the emergence of the γ-burst, they found increased activity in the alpha (α) band (~10 Hz) over the right parieto-occipital cortex. While α-based activity has been postulated as a correlate of the internal elaboration of task-related information crucial for the subsequent achievement of an *Eureka!* moment, the switch between α and γ activity has been promoted as the basic process triggering a successful insight moment (see Fig. 1a). Despite a growing body of fMRI and EEG evidence, the neurophysiology of insight processes is still in its infancy. Perhaps, one of the most important limitations is that the evidence connecting neurophysiological substrates and problem-solving processes is necessarily correlational, thus not able to offer any clues for causal interpretations. Here we tried to address this issue by directly modulating the activity of specific brain regions using Noninvasive Brain Stimulation (NiBS^17,18^) thus inducing potentially relevant changes in brain activity responsible for measurable changes in behavior. Based on previous knowledge, we investigated the role of α (10 Hz) and γ (40 Hz) oscillations constantly delivered via transcranial alternating current stimulation (tACS) during different insight problem-solving tasks, *i.e*., the (i) Compound Remote Associate problems (CRA, verbal insight^19,20^) and the (ii) Rebus Puzzles (visuo-spatial insight^20^), in a cross-over, double-blind placebo-controlled study. Recent studies suggest the feasibility of frequency-specific interactions between externally applied sinusoidal currents and endogenous brain oscillations: simulations, supported by empirical evidence using EEG, demonstrate that the very basic mechanisms by which tACS modulates brain oscillatory activity is through network resonance, with cascade effects causing large-scale, frequency-specific modulations of oscillatory network activity^21^. Animal models have documented how tACS might be able to entrains neurons in widespread cortical areas^22^, while emerging experimental evidence is showing how the effects of weak electric fields applied on optogenetically-controlled slices of pyramidal cells are constrained by their own endogenous cortical oscillations^23^. As a consequence of this externally induced modulation, effects have been documented at the behavioral level in the sensorimotor^24–26^, visual^27^, somatosensory^28^ as well as higher-order cognitive domains^29–31^ in humans.

We reasoned that: (i) a tACS-induced frequency-specific modulation of endogenous brain oscillations will produce changes in individual performance during insight problem-solving, *i.e*., higher accuracy levels; (ii) more specifically, tACS in both the α and γ bands will enhance the performance of healthy individuals solving problems via insight processes, based on available regional oscillatory evidence^6^; (iii) stimulation will also modulate individual self-report measures of performance, specifically affecting the number of answers to CRA and Rebus Puzzles participants attribute to insight rather than analytic processes.

Finally, in order to capture cognitive and brain activity factors affecting the response to NiBS, we also recorded EEG before and after each stimulation session, looking for spectral power changes induced by tACS, possibly reflecting behavioral effects. Moreover, given that individual responses to NiBS might be linked to differences in cognitive profile and brain connectivity patterns^32^, resting-state fMRI data and cognitive scores were also collected and correlated with the behavioral enhancement by tACS.

## Results

### Statistical design and behavioral data analysis

Accuracy and reaction times (RTs) for correct responses were collected for both CRA and Rebus Puzzles tasks. Analyses were carried out using IBM SPSS Statistics (Version 21, release 21.0.0) and MATLAB (Release 2012b, Mathworks). Data were filtered for outliers (mean ± 2 SD of accuracy and RTs values, respectively 4% and 3% of the overall trials). A repeated measures ANCOVA was used to investigate main effects and interactions of (i) Stimulation (Sham, tACS 10 Hz, tACS 40 Hz), (ii) Task (CRA, Rebus Puzzles) and (iii) Solution Style (Insight, Analytical method), with within-subjects factors for both correct accuracy and correct RTs. Gender, age and the order of stimulation conditions were added as covariates. In the event of a significant effect of stimulation, further simple main effects were analyzed using a similarly structured ANCOVA to decompose each effect. In the event of an interaction between stimulation and task type and a subsequent significant simple main effect of stimulation on a specific trial type, pairwise comparisons were performed to elucidate the nature of the effect. In the event of a violation of Mauchly’s test of sphericity, we employed multivariate measures. For all tests the level of significance was set at p ≤ 0.05 and multiple comparisons were corrected using Bonferroni corrections.

### CRA and Rebus Puzzles

#### Accuracy

**Figure 2:**
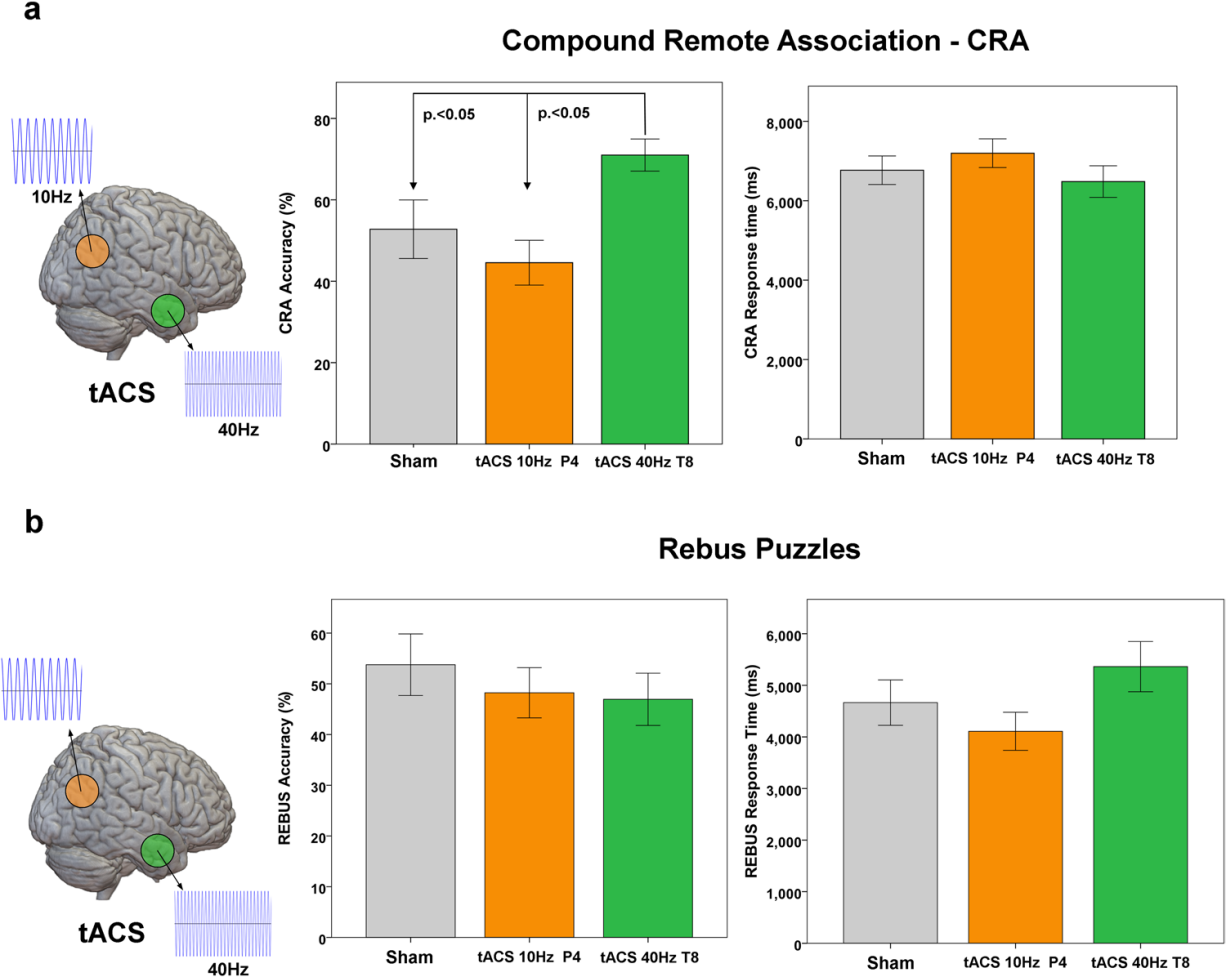
Behavioral results. Accuracy and response times for correct answers are reported for both tACS and Sham conditions, respectively for CRA (**a**) and Rebus Puzzles (**b**) tasks. Standard error values are also reported. Statistical results refer to an ANCOVA model including age, gender and stimulation order as covariates (Bonferroni corrected, see Supplementary Information for details). Lines represent standard errors of mean.

On average, during the Sham condition participants solved correctly 8.25 (SD 2.8) CRA trials, equal to 55% of the given problems. Of these, 5.2 (~63%) were solved via Insight and 3.05 (~37%) via Analytical reasoning. For the Rebus Puzzles, participants solved 5.94 (SD 2.4) trials, equal to 54% of the given problems, of which 3.1 (~52%) were solved via Insight and 2.84 (~48%) via Analytical reasoning. Statistical analysis revealed a significant effect of Stimulation [F_(2,28)_ = 4.75, p < 0.001] and Task type [F_(1,29)_ = 4.12, p < 0.01], as well as a significant Stimulation*Task type interaction [F_(2,29)_ = 3.97, p < 0.01]. A trend toward significance for Solution Style was also found [F_(1,29)_ = 2.39, p = 0.096]. Regarding Stimulation effects, tACS at 40 Hz was significantly different than tACS at 10 Hz [t_(30)_ = 3.17, p < 0.010] and Sham stimulation [t_(30)_ = 3.54, p < 0.01]. In particular, the Stimulation*Task type interaction showed a significant effect for tACS at 40 Hz over the temporal lobe during CRA [t_(30)_ = 5.98, p < 0.0001], with no effect on Rebus Puzzles trials [t_(30)_ = 0.49, p = 0. 412] (Fig. 2). tACS at 10 Hz was not significantly different than Sham [t_(30)_ = 0.34, p = 0. 512].

**Figure 3:**
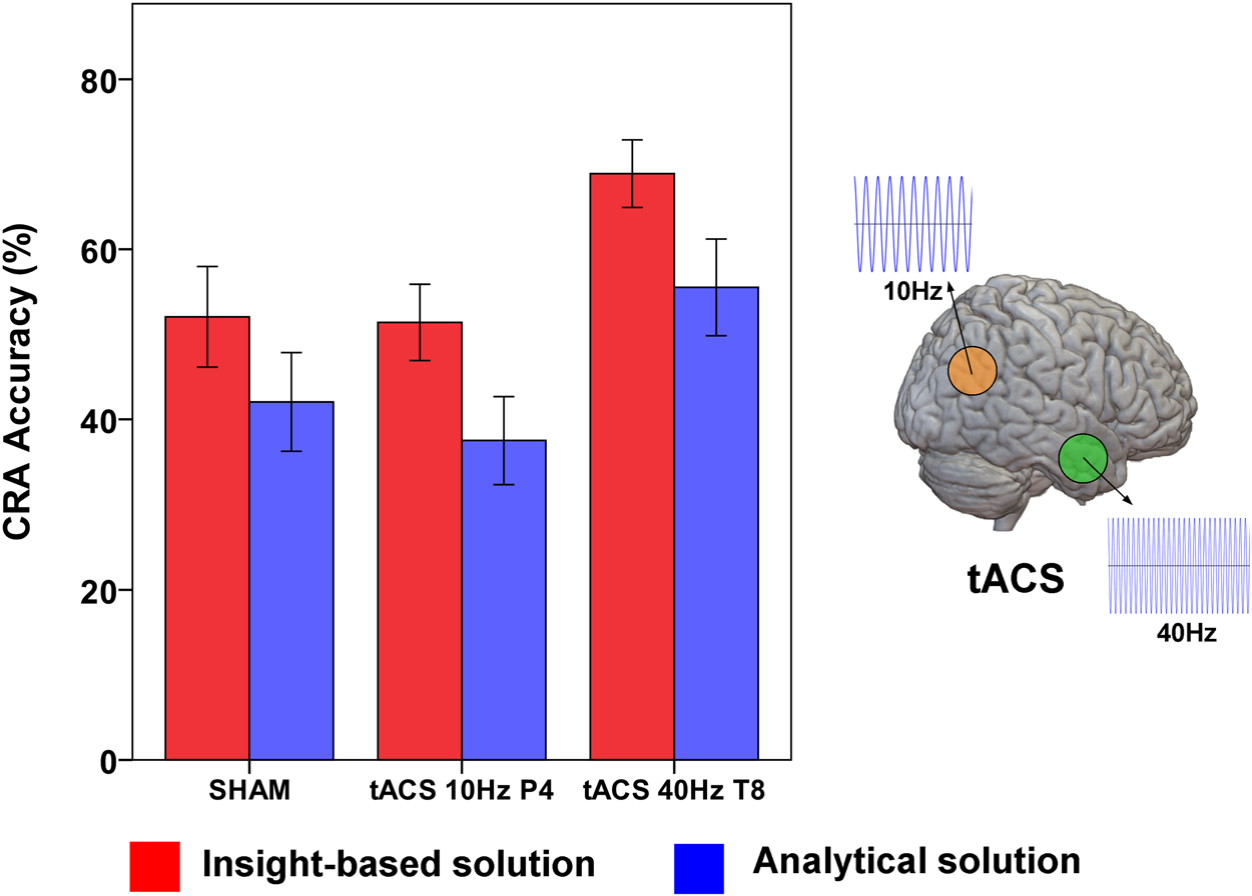
tACS effect on Individual problem-solving strategies in CRA. Stimulation at 40 Hz over the right temporal lobe induced an increase in accuracy levels at the CRA task, with no statistically significant differences for the accuracy of trials solved via Insight or Analytical problem-solving.

The average increase in accuracy during γ-tACS over T8 compared to Sham condition was 19%, while it was 27% compared to α-tACS over P4. As mentioned above, the simple main effect of Solution Style, *i.e*., the personal evaluation of each participant about her/his own responses being the result of an insight- or analytic-based problem-solving process, showed a marginally significant effect [F_(1,29)_ = 2.79, p = 0.066]. The increase in accuracy observed for tACS at 40 Hz seems to be associated with a slightly higher increase in the number of answers achieved via Insight respect to Analytical processing (Fig. 3).

#### Reaction Times

Mean reaction times of correct responses are shown in Fig. 2. Statistical analysis did not reveal any significant effect for Stimulation [F_(2,28)_ = 1.43, p = 0.328], Task type [F_(1,29)_ = 1.21, p = 0.545] and Solution Style [F_(1,29)_ = 1.671, p < 0.297]. No significant interaction was found.

### Control task

Analyses of the odd/even task revealed significant main effects of the Order in which blocks were presented over RTs [F_(3,81)_ = 3.46, p < 0.05]. Pairwise comparisons revealed that the only significantly different block was the first one [block 1 vs. block 2: t_(23)_ = 3.47, p < 0.05; block 1 vs. block 3: t_(23)_ = 2.19, p < 0.05; block 1 vs. block 4: t_(23)_ = 2.10, p < 0.05, all other pairwise comparisons were not significant (p > 0.2). No effects for Accuracy were found [F_(3,81)_ = 3.46, p > 0.05]. The same analyses were also performed with blocks ordered by the stimulation type they followed, an important control that could detected whether any of the stimulation types had general after-effects on RT or accuracy levels. No significant differences were observed on RT and accuracy [RT: F_(3,92)_ = 3.89, p > 0.05, Accuracy: F_(3,92)_ = 4.69, p > 0.01].

### EEG

**Figure 4:**
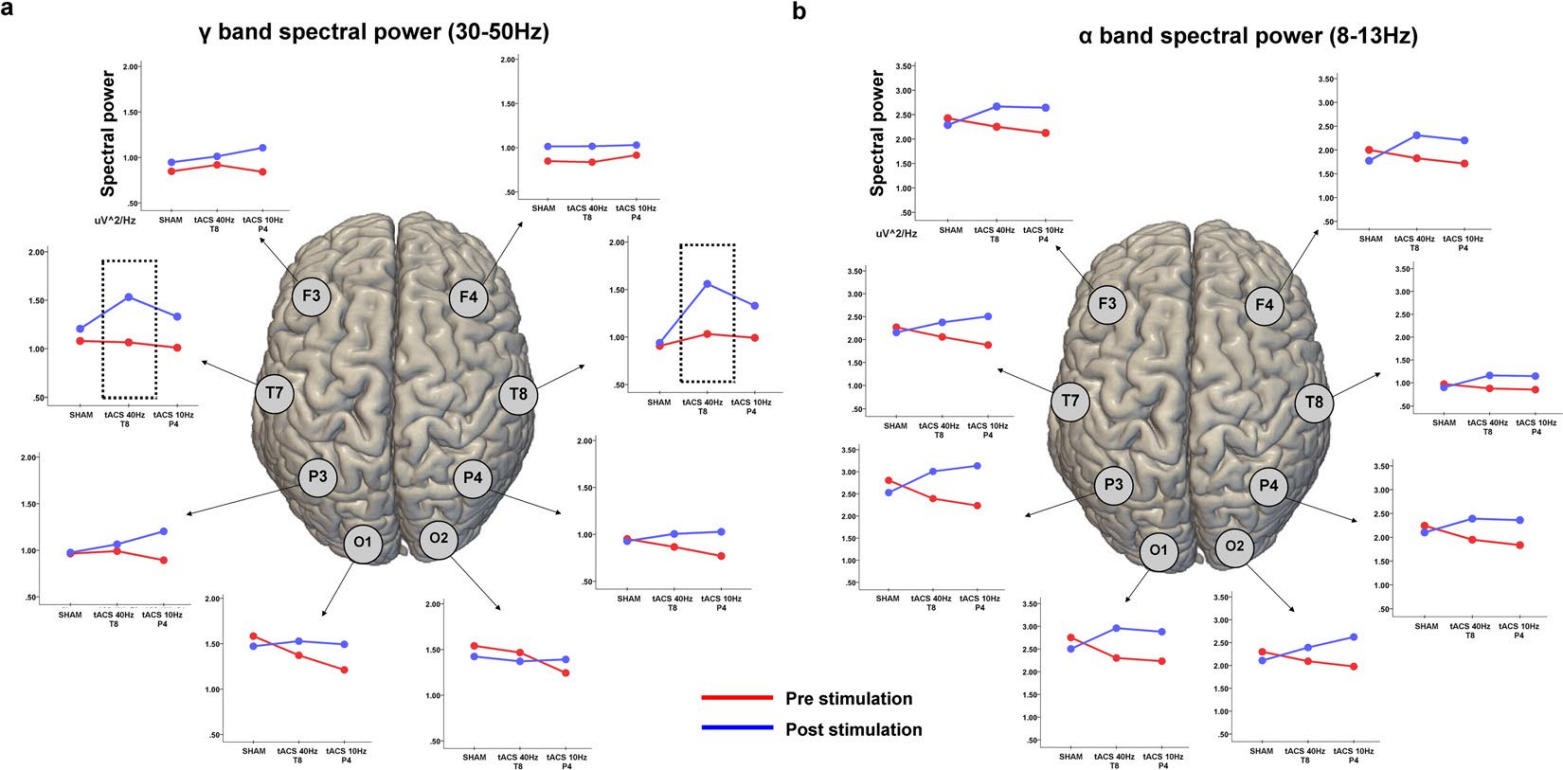
EEG changes after tACS. Line plots represent the average spectral power in the low-γ (30–45 Hz) (**a**) and α (8–12 Hz) (**b**) frequency bands before and after stimulation, for each recording electrode. Values are reported for each stimulation condition, including Sham. An increase in the power of γ oscillations over T8 (i.e., the site of stimulation) and T7 (i.e, the homologous region in the contralateral hemisphere) was found after 40 Hz-tACS, while a generalized increase in α power is present for almost all the electrodes, possibly due to increase drowsiness.

Analyses revealed a significant effect of Stimulation [F_(2,28)_ = 6.73, p < 0.01] for spectral power of the γ band (i.e. 30–50 Hz). No significant main effects were found for spectral power computed within the θ [F_(2,28)_ = 0.345, p = 0.498] and β [F_(2,28)_ = 0.344, p = 0.516] bands, while a trending toward significance result was found for activity in the α band (i.e. 8–12 Hz) [F_(2,28)_ = 2.93, p = 0.098]. As for the effect of Stimulation in the γ band, 40 Hz-tACS was significantly different than both Sham [t_(30)_ = 5.73, p < 0.001] and tACS 10 Hz [t_(30)_ = 3.41, p < 0.041]. The Stimulation*Task type interaction showed a significant effect for 40 Hz-tACS on the spectral power data derived from electrodes T7 [t_(30)_ = 9.86, p < 0.001] and T8 [t_(30)_ = 10.71, p < 0.001] in the low-mid γ band (i.e. 30–50 Hz) (Fig. 4).

### Predictors of response to tACS

#### rs-fMRI

**Figure 5:**
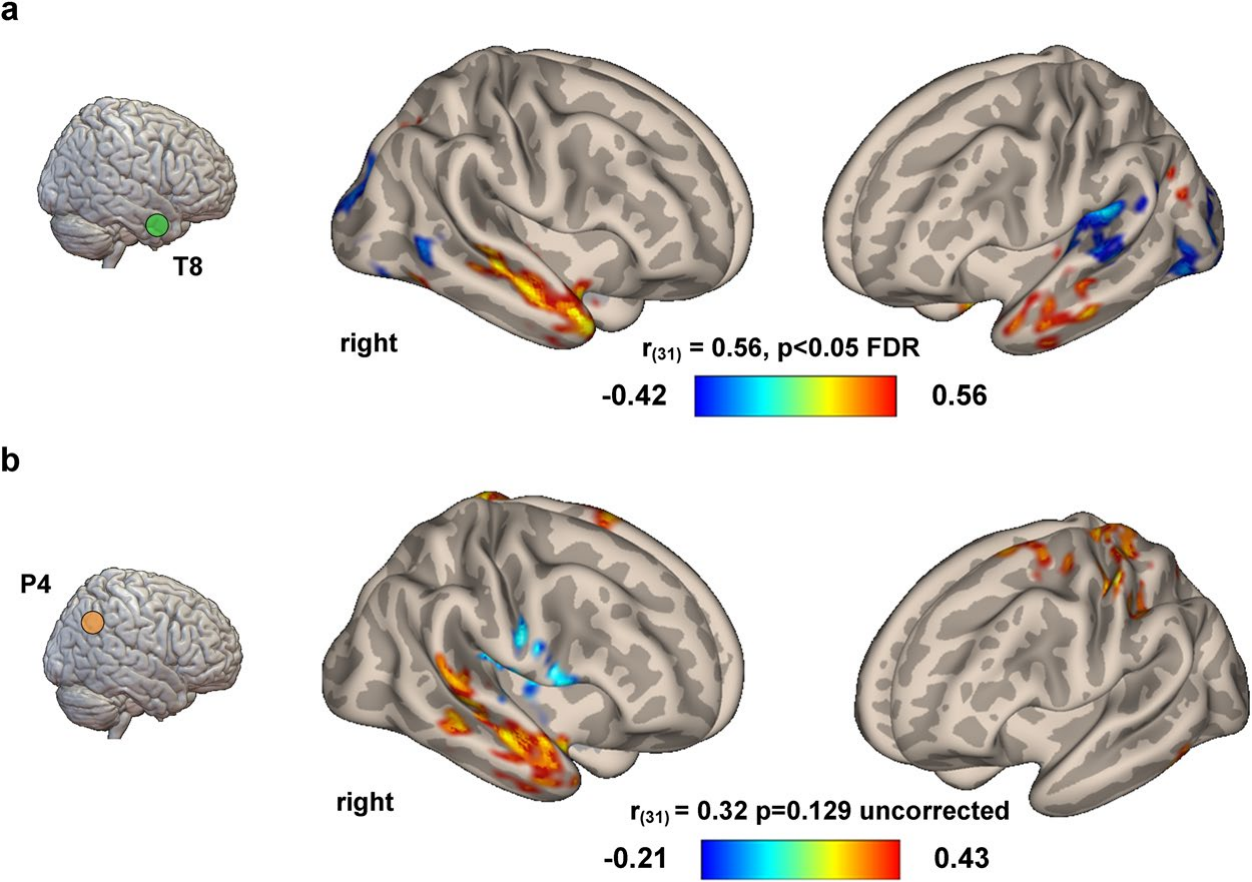
Functional connectivity correlates at rest. Resting-state data collected in all the participants were correlated with the difference in behavioral performance obtained during stimulation over the right temporal lobe (**a**) and parietal lobe (**b**). Voxel-wise seed-brain connectivity values were calculated using two anatomical ROIs representing the stimulation targets (see the Method section for additional details). The increase in performance (i.e. accuracy) observed after 40 Hz-tACS (A) at CRA was positively correlated with the strength of the connectivity between right and left anterior temporal lobes at rest, as well as the negative correlation between right temporal lobe and, respectively, bilateral occipital lobe and left supramarginal gyrus (p < 0.05 FDR corrected, p < 0.05 uncorrected at cluster-level). Additionally, panel b shows an uncorrected but interesting correlation between the behavioral response to tACS delivered on P4 and the connectivity between the same area (right parietal lobe) and the ipsilateral temporal lobe/contralateral somatosensory cortex (p. 0.001 uncorrected, p. 0.05 uncorrected at cluster level), highlighting a potential link between the two stimulation regions. Colorbars represent Pearson correlation coefficients.

Given the behavioral results suggesting tACS affecting the performance at CRA but not Rebus Puzzles, correlations between seed-based brain connectivity values (Pearson product-moment correlation coefficients) and changes in behavioral scores were computed for Delta accuracy values (i.e. Sham vs tACS) related to the CRA task only. A significant correlation between the seed-based brain connectivity of the right anterior temporal lobe (see Supplementary Information for the MNI coordinates of the seed region) and the delta values obtained using individual accuracy levels recorded during Sham and 40 Hz-tACS conditions was found [r_(31)_ = 0.56, p < 0.05, FDR corrected] (Fig. 5). No significant correlations were found for the connectivity of right parieto-occipital region [r_(31)_ = 0.23, p = 0.239]. As for delta values referring to 10 Hz-tACS, no significant correlation was found for the connectivity of the temporal lobe seed, while a trending to significance result was found for connectivity of P4 site [r_(31)_ = 0.38, p = 0.129], even though without correction for multiple comparisons at both voxel and cluster levels (Fig. 5b). We decided to report this result in Fig. 5b since the highlighted connectivity pattern is suggestive of the fact that an increase in the connectivity between right parietal and right temporal lobe (the two stimulation sites) may be responsible for fluctuations in the performance at CRA task during 10 Hz-tACS as compared to Sham.

#### Individual cognitive scores

**Figure 6:**
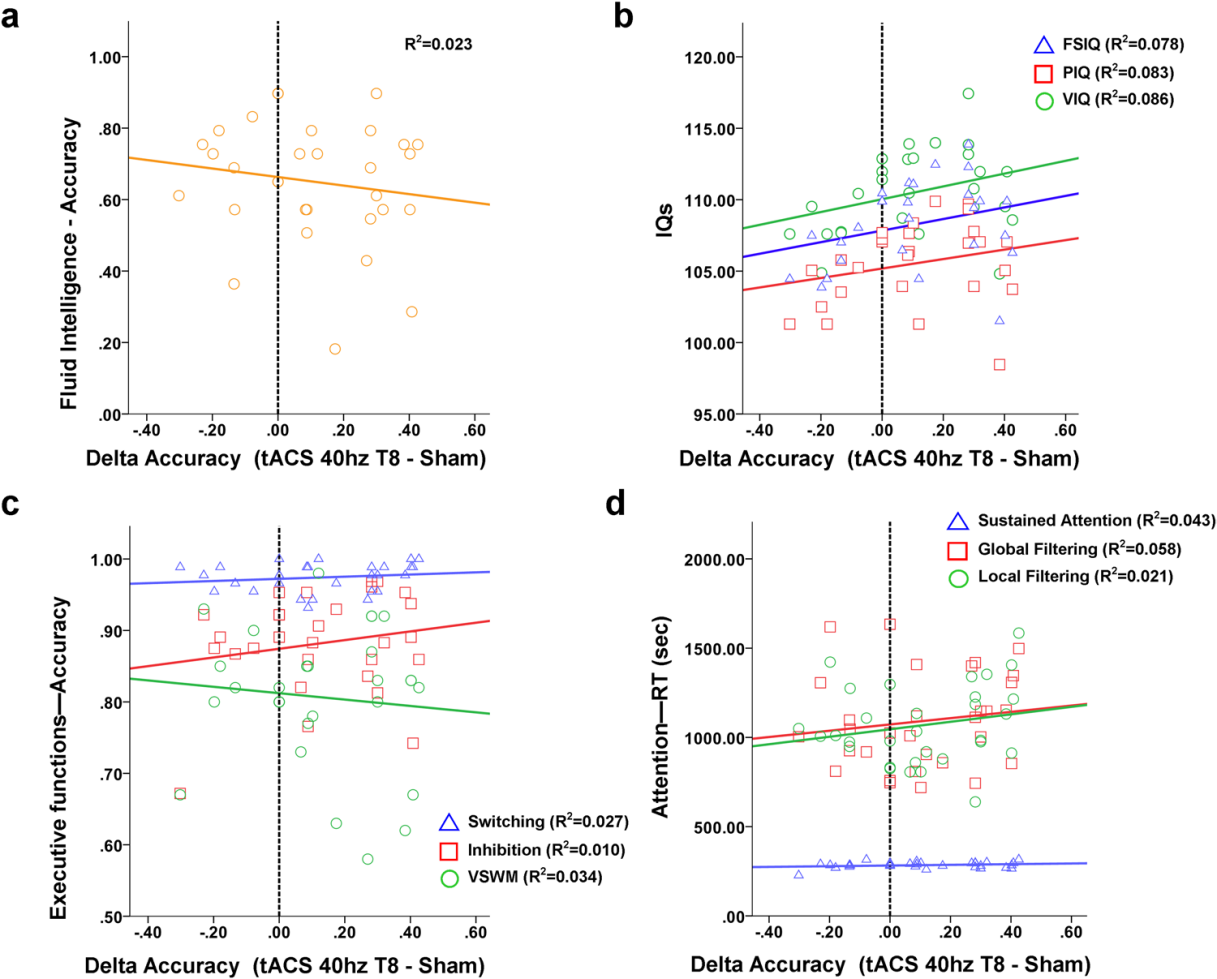
Cognitive factors related to responsiveness to tACS. Correlations between cognitive scores encompassing different cognitive domains and the changes in accuracy observed during tACS at 40 Hz over the right temporal lobe. Results specifically refer to (**a**) abstract reasoning/fluid intelligence abilities, IQ (**b**), executive functions (**c**) and attention (**d**). Details about each cognitive task are included in the Supplementary Information of the manuscript.

As shown in Fig. 6, the correlations between individual performance at tasks exploring different cognitive domains and the increase in accuracy during 40 Hz-tACS over the right temporal lobe did not show any significant value. Single correlation values refer to delta in accuracy level during tACS and: Full-Scale Intelligence Quotient (FSIQ, r = 0.256; p = 0.165); Performance Intelligence Quotient (PIQ, r = 0.231; p = 0.212); Verbal Intelligence Quotient (VIQ, r = 0.327; p = 0.096); fluid intelligence (r = −0.150; p = 0.420); Inhibition (r = 0.132; p = 0.488); Switching (r = 0.162; p = 0.385); Visuo-Spatial Working Memory (r = −0.125; p = 0.511); Sustained attention (r = 0.004; p = 0.983); Global filtering (r = 0.144; p = 0.440); Local filtering (r = 0.031; p = 0.869); Digit span forward (r = 0.083; p = 0.658); Digit span backwards (r = 0.098; p = 0.602). For details about the cognitive task, see Supplementary Information.

## Discussion

Research on the neurophysiological correlates of insight problem-solving is still at its early stages^6,33^: correlational evidence suggests a possible role of specific oscillations in the right parieto-occipital (in the α band) and temporal pole regions (in the γ band), according to the particular problem-solving style adopted. The current study is a novel attempt to causally test the role of α and γ oscillatory activity in these regions, showing a modulation of individual problem-solving abilities during the application of frequency-specific noninvasive electrical stimulation. Both participants and investigators were unaware of the type of tACS they were receiving/delivering and thus blinded to the predicted outcomes. Overall, tACS at 40 Hz over the right temporal lobe improved the accuracy at CRA by about 20%, with no significant effects on accuracy for Rebus Puzzles, while reaction times for both CRA and Rebus Puzzles were unaffected by tACS. Improved accuracy during γ-tACS was paralleled by a significant increase in the spectral power within the low-mid γ (30–50 Hz) frequency band. This effect, which was observed selectively during 40-Hz tACS, suggests an increase in fast oscillatory neuronal activity as a potential substrate for the observed behavioral results. Moreover, resting-state fMRI connectivity analyses suggested a significant positive correlation between the connectivity of bilateral temporal regions at rest and the cognitive enhancement induced by 40 Hz-tACS, while individual cognitive profile did not account for such modulation of performance.

Even though the effects of tACS in the γ frequency band were reasonably expected based on previous correlational evidence (for a review see^6^), the null effects for stimulation in the α band and on Rebus Puzzles open up additional questions. There is a general agreement that high frequency synchronization plays an important role in the large-scale coordination of activity relevant for cognition^34,35^, although there is some controversy over the potential artifactual nature of fast oscillation recordings in humans^36^. We speculate that γ activity might be involved in the multistep process behind the emergence of insight solutions, considering its involvement in a wide range of cognitive processes (*e.g*., attention^37^, memory^38^, language^39^, learning^40^, as well as with basic stimulus-response processes^41^, neural binding^41^ and cognitive control^42,43^). Indeed, a burst of γ activity has been documented as occurring in the right temporal lobe right before participants solve a problem via insight processes^8^, and even during intensive cognitive effort in chess players^44^. Interestingly, previous neurophysiological evidence has specifically linked γ-band oscillations with the temporal processes required for the activation of a mental representation^37,45^ during the emergence of integrated solutions to consciousness^46^. Therefore, the γ burst identified just before the emergence of insight solutions could well represent the transition of a new solution to consciousness. Previous noninvasive brain stimulation studies involving γ activity have causally demonstrated how tACS tuned to this frequency band might lead to increase of cognitive performance related to perception^47^ and fluid intelligence/abstract reasoning^29,30^. Although no direct evidence of the so-called “entrainment” of endogenous oscillations by means of tACS is available in humans due to intrinsic limitation of cortical recording during transcranial stimulation, both animal^22^ and *in-vitro*^48^ studies have provided support for this mechanism of action. The EEG results of the current study, showing increased power in γ range after 40 Hz-tACS, are also in line with this hypothesis. Importantly, the high-frequency nature of the stimulation applied in the present work might suggest the possibility of a frequency-unspecific effect based on the injection of “noise” instead of 40 Hz oscillatory patterns, mediated by signal degradation due to the transcranial nature of stimulation. However, recent research comparing the effect of tACS and noise-based transcranial stimulation on cognition has confirmed a differential behavioral effect for the two approaches^29^. Also, EEG data collected after stimulation confirmed a local tACS effect on γ oscillations, to a certain extent limited to the stimulated region.

Our results showed no effects for stimulation in the α frequency range, even though the model by Jung-Beeman (see Fig. 1a) would have predicted a possible effect for such stimulation on the right parietal lobe. Contrary to the well-defined fMRI and EEG evidence regarding the role played by the right temporal lobe during insight problem-solving, less EEG evidence is available for parietal/parieto-occipital activity during *Eureka!* (see^6,33^). This might have led to a less precise optimization of the applied electrical field, and thus to a less effective stimulation pattern (see Supporting Information for modeling of induced electric field). Moreover, while a role for right temporal lobe activity in processes relevant for insight problem-solving has been demonstrated by multiple language studies focusing on the integration of distant or novel semantic relations during language comprehension^49,50^ and coarse semantic coding^6,12^, the role of the right inferior parietal lobule has been less characterized. This area seems to be implied in revealing false semantic relations on provided statements, suggesting its involvement on inference and inhibition processes necessary for determining semantic coherence^51^, a process which might fit with the first step of Beeman and colleagues’ model (i.e. inhibition of problem’s initial representation). However, the anatomical definition for such evidence is less precise, and activity in the occipital cortex and contralateral –left— parietal lobe has been proposed as well (for a review see^6^). In addition, it may be possible that while α activity in the right parietal lobe is indeed necessary for insight processing, it is not the main determinant – that is, it makes insight processing possible but does not ensure it will occur. Finally, α oscillations were reported to reflect the inhibition of cortical areas^52^, therefore, α activity before an *Aha!* moment could represent the inhibition of activity in the visual cortex^8^. Interestingly, eye movement data shows that insight problem-solving is associated with an interruption of visual input^53^. Such inhibition might limit the external information flow that could interfere with high-demanding top-down processes supporting the reconstruction of the information at hand^54–57^, thus widening the potential anatomical candidates to be targeted with α stimulation. Future studies might examine the use of tACS over the occipital cortices or a more widespread network including both parietal and occipital regions.

Despite the focus of our tACS protocol on the right hemisphere, both the increase of γ power (Fig. 4) and the positive correlations between the strength of fMRI connectivity with behavioral data (Fig. 5) were detected in bilateral temporal regions. Previous investigations suggest distinct roles for the two hemispheres during insight problem-solving. In a set of experiments aimed at elucidating the role of hemisphere-dependent processing, Bowden and Beeman used a visual-hemifield presentation of the CRA^9^. As a result, subjects showed more solution priming when recognizing solutions with a feeling of sudden insight than without it, also showing an increased in the total number of correct solutions when stimuli were presented in the right visual hemifield compared to left one. These results demonstrate the role played by the right hemisphere in semantic coding and the activation of alternative meanings. On the other hand, the left hemisphere engages in fine semantic coding, specifically activating a single interpretation and only a few close or contextually appropriate alternatives^10–12^. In a similar way, insight problem-solving requires alternative interpretations of chunks of information that initially appear unrelated to the problem and should mostly benefit from coarse semantic processing happening in the right hemisphere^9,14,58^. Originally, our data show a bilateral network of regions, mostly belonging to the temporal lobes, as being correlated with the behavioral effects, both in terms of an increase of γ oscillations after stimulation (EEG) and as a positive correlation with their fMRI functional connectivity at rest. Effectively, Tian *et al*.^59^ suggested a bilateral model in which a first activation in the left hemisphere (i.e. temporal and prefrontal regions) supports the preparation for insight, while a subsequent right temporal activation leads to the *Eureka!* moment. Additionally, prior investigations using transcranial Direct Current Stimulation (tDCS) have shown some evidence of the potential value of stimulating regions in both hemispheres. Cerruti and colleagues^60^ reported that anodal stimulation of the left dorsolateral prefrontal cortex (and cathodal stimulation of the right supraorbital region) enhances problem-solving in the Remote Associates Task (RAT)^60^. Chi *et al*.^61^ found that anodal stimulation over the right anterior temporal lobe and cathodal stimulation of the left anterior temporal lobe increases performance in the Matchstick Arithmetic Task^61^. In a follow-up experiment^62^, the same group confirmed that anodal stimulation of right temporal lobe (coupled with cathodal stimulation of the contralateral one) affected the performance at the nine-dot problem, while the opposite electrode montage was not effective (i.e. anodal on the left lobe and cathodal on the right one). More recently, Aihara and colleagues (2017) showed no significant effect on RAT and Matchstick task for anodal tDCS over the right anterior temporal lobe. However, the study targeted a slightly different site (T4) and used very large conductive rubber electrodes (7 × 5 cm^63^), known for inducing a very widespread cortical field as compared to the more focal montage based on small circular electrodes used in the present study. Finally, Ruggiero and colleagues showed reduced reaction times with anodal tDCS over the left temporal lobe (and cathodal over the right one) during RAT^64^.

Although previous studies adopted different types of tES simultaneously affecting multiple regions with both inhibitory and excitatory stimulation (i.e. cathodal and anodal stimulation), as well as two tasks which have since been questioned for their validity when assessing insight^7^, overall they suggest that there may be an effect for left-lateralized or bi-hemispheric stimulation protocols as well, in line with our EEG and fMRI findings. However, it must be considered that tACS operates following different principles than tDCS and it might be difficult to reconcile previous and current results in the context of inhibition/excitation of brain regions. More specifically, tACS is based on oscillatory potentials resulting from a fast switch of polarity across scalp electrodes, defined by the stimulation frequency. In the case of 40 Hz stimulation, for instance, electrodes are initially assigned a given polarity (either positive -anode- or negative -cathode-) and then continuously alternate between the two polarities 40 times per second. This generates an oscillatory field able to entrain local neuronal activity at the specific stimulation frequency, without inducing specific inhibitory or excitatory effects due to stimulation polarity as in the case of tDCS. Therefore, the impact of bilateral temporal or prefrontal tACS might not reflect what previously observed with bilateral tDCS. Moreover, it must be considerd that tACS seems less likely to induce carryover effects compared to tDCS^65,66^. To further control for offline effects in the current study, a control task and a resting period were introduced in-between the tACS blocks. Additionally, the order of stimulation conditions was counterbalanced across subjects and included as covariate in the statistical analyses. However, systematic investigations are needed to compare the online/offline effects of tDCS and tACS over bilateral temporal lobes as well parietal and prefrontal regions.

A current debate is whether insight performance should be ascribed to the overall accuracy at a given insight-related task, or instead should correspond to the subjective feeling of having a *Eureka!* moment (e.g. not being able to explain your own problem-solving strategy). As a consequence, enhancement of insight abilities might be seen either as the attempt to increase (i) one’s pure performance (i.e. accuracy) at a given insight task, or (ii) his/her awareness of a given answer being achieved via a *Eureka!* moment or not. In our experience, tACS has been able to increase individual performance at a specific insight task, with also a trend towards an increased number of solutions achieved via a problem-solving process subjectively labeled by participants as a *Eureka!* moment. So, should the task adopted for the present study be the criteria to decide if we enhanced insight problem-solving, or did we simply increase general problem-solving abilities? In this regard, it is worth consider that findings by Beeman and colleagues demonstrate how sudden insight occurs when people engage distinct neural and cognitive processes that allow to see connections that previously were eluded^7^. Insight problem-solving is a discrete process that relies on bursts of awareness of weakly activated concepts remotely associated to the elements of the problem (thus not easily describable in terms of a sequence of logical reasoning steps towards the correct solution to a problem), while analytical problem-solving is a continuous step-by-step reasoning process completely available to consciousness (*e.g*.^6,8^). In the context of our results, we speculate that the driving force of the effect observed in insight problem-solving could be controlled by right superior temporal gyrus activation. Specifically, 40 Hz-tACS might have induced two effects that are not mutually exclusive. On one side, assuming γ activity is representing the actual process needed to organize available inputs into a meaningful answer, tACS might have enhanced computational processing efficiency, therefore inducing an effect on general problem-solving skills (i.e. affecting both analytical and insight strategies). On the other hand, assuming γ is responsible for the switch between subconscious and conscious knowledge, 40 Hz-tACS might have amplified fast oscillatory activity in the right temporal lobe, thereby increasing participants’ awareness of their reasoning steps while also connecting weakly activated information. While these two effects might coexist, an increased number of solutions obtained/labeled via analytical reasoning should have been observed following 40 Hz-tACS, whereas our data show a slightly higher increase in solution given via insight. Unfortunately, our experimental design, as well as insight-related tasks available in the literature, do not allow for a full dissection of this issue, which is actually crucial for the success of future neuromodulatory interventions and possibly requires the identification of new assessment tools.

Whereas the accuracy in the CRA was substantially improved by γ band-tACS, the accuracy in the Rebus Puzzles was not significantly affected by any of the stimulation conditions. It is worth noting that the performances at the CRA and Rebus Puzzles showed only a weak correlation with each other (correlation between accuracy values across all stimulation conditions, r = 0.24, p = 0.358), suggesting the two tasks do not measure the exact same constructs, and possibly do not activate the same neurophysiological substrates. Additionally, although both CRA and Rebus Puzzles can evoke insight solutions, these tasks rely on different types of stimuli, i.e. verbal *vs*. visuo-spatial ones, and therefore their solutions could involve different processes. Whereas it is reasonable to postulate a more semantically-related process for the CRA, a more widespread activation of regions in the temporal, parietal and occipital lobes could support the solutions for Rebus Puzzles, making our focal stimulation solutions somehow ineffective. Unfortunately, while neuroimaging data are available for CRA, RAT and anagrams^8,67,68^, no data have been reported for purely visual insight stimuli like the Rebus Puzzles, making it difficult to tailor more appropriate tES solutions. Beeman and Bowden hypothesized the existence of a shared network of brain regions responsible for insight problem-solving regardless of the nature of the stimuli at hand^69^. This is somehow in contrast with the present findings and suggests the need for larger investigations that also include the recording of event-related neurophysiological data (e.g. EEG) before, after and possibly during stimulation.

Our stimulation approach provides a constant delivery of oscillatory currents, which might have affected brain local spike timings through the entire stimulation sessions, i.e. regardless of the two-steps process formalized by Jung and Beeman (i.e. switch between α and γ activity right before an insight solution). This might have led to undesired increases of α and γ activity during, respectively, the second and first part of the hypothesized multistep insight process, therefore reducing the effectiveness of tACS instead of promoting cognitive enhancement. Close-loop stimulation solutions might be implemented^70^, allowing for online monitoring of individual brain oscillations during problem-solving and consequent dynamic triggering of γ/α tACS bursts. Moreover, previous investigations using a different form of tES, which do not probe specific brain oscillatory patterns but rather modulate cortico-spinal excitability (i.e. tDCS^71,72^), have suggested a role for the excitability of the bilateral temporal lobes^61,62^ and the left prefrontal cortex^60,73^. Further investigations addressing the role of specific brain oscillations in these regions using tACS are needed, as well as testing the possibility of using random-noise stimulation (tRNS) to increase local cortical excitability^74^.

Interestingly, several behavioral/cognitive factors have been shown to be correlated with insight-related performance^6^. However, our data do not support a role for individual cognitive profiles in determining individual response to tACS. Additionally, we did not check for additional potentially confounding factors. For instance, positive mood seems to influence the tendency of utilizing insight instead of analytical problem-solving during CRA^75^, mindfulness scores were found to correlate with performance on Rebus Puzzles^76^, time of the day seems to be related to performance on insight-related tasks with participants solving trials more efficiently during their non-optimal time of the day (suggesting a potential beneficial role for the decrease in inhibitory prefrontal control)^77^. Future studies should address the role of these measures in explaining variability in the response to tES during insight problem-solving.

In conclusion, our data provide a first evidence of the causal role of γ oscillations in the right temporal lobe for the genesis of *Eureka!* moments in humans, also suggesting its susceptibility to external modulation.

## Methods

### Participants

Thirty-one healthy subjects (17 female, age 24.4 +/− 3.8 years) participated in the study after giving their written informed consent. All procedures were performed in accordance with relevant research guidelines and regulations, and approved by the Regional Ethical Review Board in Siena (Italy). All the participants were healthy, native Italian speakers. Thirty participants were fully right-handed, one was left-handed as assessed by the Oldfield Handedness questionnaire^78^. Their neurological and psychiatric examinations were normal. Exclusion criteria also included the use of drugs or illicit substances acting on the central nervous system in the days preceding the experiment.

### Experimental procedures

Each participant completed an experimental session composed by 3 blocks of each insight task (i.e. Compound Remote Associates problems –CRA— and Rebus Puzzles) while receiving tACS or Sham (i.e. placebo) stimulation. Stimuli and instructions were presented using E-prime 2.0 software (Psychology Software Tools, Inc., PA, USA). Participants were comfortably seated in a quiet room, positioned at about 50 cm from an LCD screen, wearing insulating headphones. Subjects performed a training session before starting the actual experiment, solving a few examples of CRA and Rebus Puzzles trials (Fig. 1). They were also instructed on the definition of a problem solution achieved via insight (*i.e*., a solution which cannot be explained in terms of a sequence of logical deductions and obtained without a clear awareness of how it has been produced) or via analytical method (*e.g*., the solution is the result of a step-by-step process based on an overt association between the given stimuli, so that the participant was able to describe the different solutions he/she discarded before getting to the right one). At the end of each trial, and after reporting the solution to the experimenter, participants were asked to specify their adopted problem-solving strategy. Stimuli were presented in the center of the display for 20 sec and participants were instructed to provide answers as accurately and quickly as possible by pressing the spacebar on a PC keyboard. After a spacebar-press or 20 seconds—whichever occurred first—a text window appeared asking the participants to input their answer. Then, a second window appeared with the instruction to press “I” or “A” to indicate how the problem was solved (via Insight or via Analytical method, respectively). After each stimulation block, subjects performed an odd-even reaction time task to assess vigilance levels.

### tACS and EEG recordings

Transcranial alternating current stimulation (tACS) was delivered using a Starstim Neurostimulator (Neuroelectrics, Barcelona, Spain) at an intensity of 2 mA peak-to-peak. As shown in Fig. 1a, the theoretical model as well as the EEG evidence proposed by Jung-Beeman *et al*.^8^, suggest a switch in the oscillatory patterns recorded right before an *Aha!* moment, highlighting the pivotal role of α oscillations in the right parieto-occipital region, and γ oscillations in the right temporal pole. Therefore, in order to test for the functional relevance of such oscillatory activity, tACS was optimized to target the right parietal lobe (roughly corresponding to electrode P4 in the 10–20 EEG system) and temporal pole (electrode T8) at 10 Hz and 40 Hz respectively. In order to maximize the focality of each stimulation pattern, a multifocal approach was adopted^79^, using an array of up to 8 stimulation electrodes placed on the scalp to increase current injection on a given cortical target. In order to keep the current intensity in the target electrode fixed (T8 for tACS at 40 Hz and P4 for tACS at 10 Hz), three electrodes were positioned on the following locations and given 1/3 of the stimulation intensity directed to T8/P4: for tACS at 40 Hz, electrodes on P3, T7, F3; for tACS at 10 HZ, electrodes on F4, P3, T7. This ensured the maximal current density in the target regions with very low intensity stimulation being delivered on other sites, therefore theoretically reducing the efficacy of stimulation on the contralateral hemisphere. The two stimulation templates and their corresponding induced electric fields are reported in Fig. S1, additional details are available in the Supplementary Information.

Stimulation intensity was ramped up for 30 seconds, then tACS was delivered for 2 minutes while participants sat on a comfortable chair staring at a crosshair on the LCD monitor (see Fig. 1 for a graphical depiction of the stimulation montages and information about experimental design and stimuli). This initial tACS during resting-state was included to potentially facilitate the entrainment phenomenon between exogenous and endogenous oscillatory activity^80^. Each participant then solved the CRA and Rebus Puzzles tasks while receiving stimulation. Before each stimulation block (tACS + CRA-Rebus Puzzles), EEG activity (3 minutes with eyes opened) was recorded from the same electrodes used to deliver tACS (N = 4) plus four additional ones placed on the scalp in order to cover each brain lobe bilaterally (i.e. 8 channels: F3-F4, T7-T8, P3-P4, O1-O2). During Sham blocks, we applied 20 seconds of tACS using the frequency of stimulation applied in the previous tACS block. Due to the low and gentle rise of the intensity of stimulation, subjects did not feel any scalp sensation. At debriefing, subjects reported that they were blind to the frequency applied and that they were not aware of the location of the stimulation. EEG was recorded using the Starstim device at a sampling rate of 500 Hz, using an average reference during recording.

### Insight tasks

Each experimental block was composed by 15 randomized linguistic CRA problems followed by 11 visuo-linguistic Rebus Puzzles, in order of ascending difficulty. The order of CRA and Rebus Puzzles trials in each block was fixed (see Supplementary Information for additional details about each task).

### Behavioral and psychometric assessment

Individual variability in the response to NiBS has been documented for both transcranial magnetic and electrical stimulation (for a review see^32^). Different factors might play a role in such variability, including structural and functional properties of the brain, differences at the cognitive level and even genetic factors (e.g. BDNF polymorphism^81^). Therefore, in order to characterize the response to tACS in our participants, both resting-state fMRI and neuropsychological data related to multiple cognitive domains (i.e. executive functions, fluid intelligence, filtering abilities, short term memory, Intelligent Quotient) were collected on a separate day from the stimulation sessions. Details about cognitive tasks are included as part of the Supplementary Information.

## Supplementary information

Supplementary Information

## Data Availability

The datasets generated during the current study are available from the corresponding author on reasonable request.
