# Supplementary material for: Gamma tACS over the temporal lobe increases the occurrence of Eureka! moments
Source: Sci Rep. 2019 Apr 8;9:5778. doi: 10.1038/s41598-019-42192-z (PMC6453961; doi:10.1038/s41598-019-42192-z)
Supplement: Supplementary file 1 — Supplementary Information [file 41598_2019_42192_MOESM1_ESM.pdf]

Gamma tACS over the temporal lobe increases the occurrence of *Eureka!* moments

Emiliano Santarnecchi *[1,2^], Giulia Sprugnoli [2^], Emanuela Bricolo [3,4], Giulio Costantini [3], Sook-Lei Liew [5], Christian S. Musaeus [6], Carola Salvi [7,8], Alvaro Pascual-Leone [1], Alessandro Rossi [2,9] and Simone Rossi [2,9]

SUPPLEMENTARY INFORMATION

- **Linguistic - CRA problems**
- **Visuo-linguistic Rebus Puzzles**
- **EEG preprocessing and analysis**
- **MRI data acquisition and functional connectivity analysis**
- **tACS computational model**
- **Cognitive Tasks**
- **Supplementary Figure S1**

Linguistic - CRA problems

Participants first solved the Italian version of the recently validated CRA problems[1] (see Fig. 1C, for the original English version see[2]). These types of problems have been consistently used to study insight problem-solving. Self-reports differentiating between insight and analytic problem-solving are reliable and their association with numerous behavioral and neuroimaging markers are well documented in literature[2,3,4,5]. CRA presents several advantages: they are composed of a large set of trials, allowing reliable data collection; they can be solved via insight or via analytical processes, allowing a direct comparison between their respective electrophysiological activations; they require short time responses and a small visual space to be presented. Each problem is formed by three words and the solution is represented by a forth word that can be associated with the others to form a compound word (*i.e., manners, round, tennis*; solution: *table).* We selected 105 items and divided them into 7 sets of 15 items each, balanced for difficulty as well as for the method preferentially used to solve them (i.e. we considered a given trial as "insight" or "analytic" based on the preferred strategy reported by participants included in the validation study by Salvi et. al., 2015[1]). Three random blocks (= 15 trials each) were selected for each participant.

Visuo-linguistic Rebus Puzzles

Participants also solved the Rebus Puzzles, an insight task initially validated by MacGregor and Cunningham[6] and subsequently validated in Italian[1]. Unlike the CRA, this set of problems requires the integration of both visual and verbal information in order to find a common phrase fitting each item (*i.e.,* "*Cycle, Cycle, Cycle*" solution: "*Tricycle*") (see Fig. 1C). Similarly to CRA problems, they can also be solved either via insight or via analytic

processes. We divided all trials in 7 sets of 11 trials each, balanced for difficulty and for problem-solving strategy; 3 blocks (= 15 trials each) were selected for each participant.

EEG preprocessing and analysis

EEG preprocessing was performed separately for data collected before and after each stimulation block, with the examiner being blinded to the different stimulation conditions for all the preprocessing steps. First, the EEG file was imported to MATLAB (vR2014b, The Mathworks) using the EEGlab toolbox (v13.4.4b)[7] and channels were located on the scalp model using the DIPFIT plugin from eeglab. Data were bandpass filtered from 1 Hz (highpass) to 70 Hz (lowpass) using a Kaiser window FIR filter. The filter order was determined using the function *pop_firwsord* with a requested transition bandwidth of 0.2 and a maximum passband deviation of 0.001. The line noise (~50Hz) was removed using the *cleanline* function, by adopting a window length of 4 seconds, an overlap of 2 seconds, an alpha value of 0.01 and a bandwidth equal to 2. Afterwards, data were epoched with an epoch length of 1 sec, and EEG epochs with clear muscle, electrode and blinking artifacts were manually removed. Given the high similarity between neural and muscle-related gamma activity as detected by scalp EEG, special attention was put on the detection of spontaneous and tACS-modulated neural gamma, by visually inspecting each electrode data. The spectral power for each epoch in four different frequency bands (θ = 4-7.99 Hz; α = 8-12.99 Hz; β = 13-29.99 Hz; γ = 30-50 Hz) was computed using the EEGlab function *spectopo*, then averaged across epochs respectively for data collected before and after the stimulation. The average power values for each subject were then included in four different multivariate ANOVA designs, respectively looking at changes in the theta (θ), α, beta (β) and γ frequency bands. Differences in spectral power before and after stimulation were investigated for each

stimulation condition (factor "Stimulation" =Sham, tACS 10Hz and tACS 40Hz), including values for each EEG electrode (n=8) using SPSS software and a statistical significance threshold equal to $p \leq 0.05$.

MRI data acquisition and functional connectivity analysis

In order to characterize the response to tACS in different individuals by means of functional connectivity analysis, resting-state fMRI (rs-fMRI) was acquired for all participants. fMRI connectivity analyses provide a non-invasive way to examine spontaneous brain functioning, which utilizes the temporal correlation between spontaneous blood oxygen level-dependent (BOLD) signal fluctuations of different brain regions to identify broadly connected networks[8]. Here we collected both structural and functional imaging data in order to correlate individual responses to transcranial electrical stimulation (tES) with spontaneous functional connectivity patterns. The neuroimaging data included a T1-weighted Fast Field Echo 1-mm thick acquisition of the entire brain (TR/TE= 30/4.6ms, flip angle = 30.00, FOV = 250 mm, matrix 256x256, slice number = 150) and an fMRI blood oxygenation level-dependent (BOLD) T2-weighted sequence acquired during rest condition with eyes open (TR/TE= 2000/35ms, 205 scans, 27 interleaved slices). Functional image preprocessing and statistical analyses were carried out using SPM8 software (Statistical Parametric Mapping; http://www.fil.ion.ucl.ac.uk/spm/) and MATLAB 7.5 (MathWorks, MA, USA). The first five volumes of functional images of each subject were discarded to allow for steady-state magnetization. EPI images were slice-time corrected using the interleaved descending acquisition criteria, and realigned and re-sliced to correct for head motion using a mean functional volume derived from the overall fMRI scans. Subjects whose head motion exceeded 2.0 mm or rotation exceeded 1.0° during scanning were excluded. In order to obtain a better estimation of brain tissues maps, we implemented an optimized segmentation and

normalization process using the DARTEL (Diffeomorphic Anatomical Registration using Exponentiated Lie Algebra)[9] module for SPM8. Briefly, this approach is based on the creation of a customized anatomical template built directly from participants' T1-weighted images instead of the canonical one provided with SPM (MNI template, ICBM 152, Montreal Neurological Institute). DARTEL allows for a finer normalization into standard space and consequently avoids under or over estimation of brain regions' volume potentially induced by the adoption of an external template. A Hidden Markov Random Field model was applied in all segmentation processes in order to remove isolated voxels. Customized tissue prior images and a T1-weighted template were smoothed using an 8 mm full-width at half-maximum (FWHM) isotropic Gaussian kernel. Functional images were consequently non-linearly normalized to standard space and a voxel resampling to (isotropic) 3 x 3 x 3 mm were applied. Linear trends were removed to reduce the influence of the rising temperature of the MRI scanner and all functional volumes were band pass filtered at (0.01 Hz $< f < 0.08$ Hz) to reduce low-frequency drift. Finally, a CompCor algorithm was applied in order to control physiological high-frequency respiratory and cardiac noise[10].

In order to analyze fMRI and behavioral scores individual delta scores were computed (i.e. accuracy during Sham minus accuracy during Active stimulation, e.g. [Sham - tACS 10Hz]), and correlated with seed-brain connectivity patterns by using two predefined regions of interest (ROI) placed in correspondence of the regions being targeted with tACS. Two 5-mm diameter spheres were created at (46, -66, 35) and (51, -4, -25), respectively corresponding to the right inferior parietal (representing P4 EEG electrode) and right anterior temporal lobe (T8) in MNI space. The average correlation between the BOLD signal from each ROI and those of each voxel in the brain were then correlated with the aforementioned delta scores, to identify patterns of connectivity co-varying with the propensity to respond to electrical stimulation during insight problem-solving. Two subjects were discarded due to

excessive head motion in the scanner, while one subject did not complete the MRI exam due to an unpleasant feeling during the acquisition. Therefore, the final analysis was conducted on 28 subjects.

tACS computational model

To check for the degree of focality of tACS solution, two models had been created separately for tACS 10Hz over P4 and tACS 40HZ over T8. Distribution of current and normal components of generated electrical fields are reported for each montage in Fig. S1. A realistic head model based on T1-weighted and Proton Density (PD) weighted phantom MRI images of the single-subject template Colin27 was used to simulate the electric field distribution using the Stimweaver software (Neuroelectrics, Barcelona), as previously described[11]. Five different tissue types were distinguished. Isotropic conductivities were used as follows: 0.33 siemens per meter (S/m) for the scalp and grey matter (GM), 0.008 S/m for the skull, 1.79 S/m for the cerebrospinal fluid (CSF, including the ventricles) and 0.15 S/m for the white matter (WM). The plugs at the apexes of the orbits were given conductivity values equal to those of the scalp. In order to represent the conductivity of sponge electrodes soaked in saline solution, the electrodes were modeled with a high conductivity value of 2 S/m. Models suggested a more focal stimulation for tACS montage targeting T8, with a smaller in magnitude and more widespread stimulation area for P4 stimulation. This could possibly affect the behavioral results by eliciting a less specific stimulation pattern during tACS 10Hz over P4. However, the two multifocal solutions both successfully achieved the goal of maximizing the stimulation field over the target regions, with little to none effects over the rest of the brain.

Cognitive Tasks

Cognitive assessment was based on a battery of PC-based tasks, encompassing global cognitive functioning scores such as Intelligence Quotient (i.e. Fullscale IQ, Performance IQ and Verbal IQ) obtained at the TIB (Premorbid Intelligence Battery) test[12,13] and fluid intelligence (Raven Advance Progressive Matrices - RAPM[14]). Additionally, domain specific tasks were administered, including: Go-NoGo task for inhibition[15], Pop-Task for Switching abilities[16], Change detection task for visuo-spatial working memory[17], Digit span forward/backward for verbal working and short- term memory[18], Visual search task for sustained attention[19], Global and Local features task for filtering abilities[20]. All the tasks were delivered using a Windows based PC, using E-prime software 2.0. Participants completed the cognitive evaluation the day of the MRI acquisition. Accuracy and Reaction Times (for correct responses) were calculated and included in the correlation analysis.

SUPPLEMENTARY FIGURE

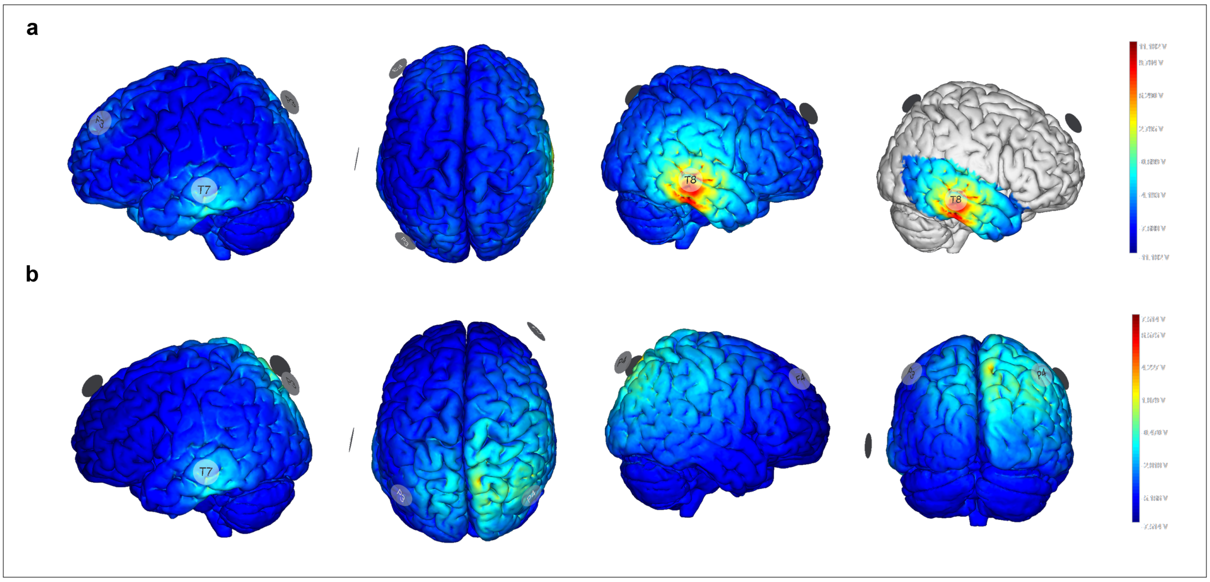

Figure S1. Surface models of the tACS montages adopted for right temporal (a) and right parietal (b) stimulation conditions. Color code refers to the amount of current delivered at the brain cortical level (Volts). Models are built using a standard head model as described in the Supplementary Information.

Reference List

1. Salvi,C., Costantini,G., Bricolo,E., Perugini,M., & Beeman,M. Validation of Italian rebus puzzles and compound remote associate problems. *Behav. Res. Methods*(2015).

2. Bowden,E.M. & Jung-Beeman,M. Normative data for 144 compound remote associate problems. *Behav. Res. Methods Instrum. Comput.* **35**, 634-639 (2003).

3. Jung-Beeman,M. *et al.* Neural activity when people solve verbal problems with insight. *PLoS. Biol.* **2**, E97 (2004).

4. Kounios,J. *et al.* The prepared mind: neural activity prior to problem presentation predicts subsequent solution by sudden insight. *Psychol. Sci.* **17**, 882-890 (2006).

5. Subramaniam,K., Kounios,J., Parrish,T.B., & Jung-Beeman,M. A brain mechanism for facilitation of insight by positive affect. *J. Cogn Neurosci.* **21**, 415-432 (2009).

6. MacGregor,J.N. & Cunningham,J.B. Rebus Puzzles as insight problems. *Behavior Research Methods* **40**, 263-268 (2008).

7. Delorme,A. & Makeig,S. EEGLAB: an open source toolbox for analysis of single-trial EEG dynamics including independent component analysis. *J. Neurosci. Methods* **134**, 9-21 (2004).

8. Biswal,B.B. *et al.* Toward discovery science of human brain function. *Proc. Natl. Acad. Sci. U. S. A* **107**, 4734-4739 (2010).

9. Ashburner,J. A fast diffeomorphic image registration algorithm. *Neuroimage.* **38**, 95-113 (2007).

10. Behzadi,Y., Restom,K., Liau,J., & Liu,T.T. A component based noise correction method (CompCor) for BOLD and perfusion based fMRI. *Neuroimage.* **37**, 90-101 (2007).

11. Miranda,P.C., Mekonnen,A., Salvador,R., & Ruffini,G. The electric field in the cortex during transcranial current stimulation. *Neuroimage.* **70**, 48-58 (2013).

12. Nelson H.E. *The National Adult Reading Test (NART): test manual*(Windsor: NFER-Nelson.,1982).

13. Sartori,G., Colombo,L., Vallar,G., Rusconi,M.L., & Pinarello,A. Test di Intelligenza Breve per la valutazione del quoziente intellettivo attuale e pre-morboso. La Professione di Psicologo 1, 2-24. 1997.

14. Raven,J., Raven,J.C., & Court,J.H. *Manual for Raven's progressive matrices and vocabulary scales.*(Oxford, UK: Oxford Psychologists Press; San Antonio, TX: The Psychological Corporation.,1998).

15. Thorell,L.B., Lindqvist,S., Bergman,N.S., Bohlin,G., & Klingberg,T. Training and transfer effects of executive functions in preschool children. *Dev. Sci.* **12**, 106-113 (2009).

16. Rosano,C. *et al.* Functional neuroimaging indicators of successful executive control in the oldest old. *Neuroimage.* **28**, 881-889 (2005).

17. Pashler,H. Familiarity and visual change detection. *Percept. Psychophys.* **44**, 369-378 (1988).

18. Wechsler,D. *Wechsler intelligence scale for children,* 1949.

19. Treisman,A.M. & Gelade,G. A feature-integration theory of attention. *Cogn Psychol.* **12**, 97-136 (1980).

20. Navon D. Forest Before Trees: The Precedence of Global Features in Visual Perception. Cognitive Psychology 9, 353-383. 1977.
